# Meta-analysis of the association of the *CYP2J2* G-50T polymorphism with coronary artery disease

**DOI:** 10.18632/oncotarget.19518

**Published:** 2017-07-24

**Authors:** Jian Chen, Dong-Fei Wang, Guo-Dong Fu, Jie Ding, Lei-Yang Chen, Jia-Lan Lv, Juan Fang, Xiang Yin, Xiao-Gang Guo

**Affiliations:** ^1^ Department of Cardiology, The First Affiliated Hospital, School of Medicine, Zhejiang University, Hangzhou 310003, China; ^2^ Pujiang Branch of the First Affiliated Hospital, School of Medicine, Zhejiang University, Cardiavascular Center of Middle Zhejiang, Jinhua 322200, China

**Keywords:** CYP2J2, G-50T polymorphism, CAD, meta-analysis

## Abstract

The association of the *CYP2J2* G-50T polymorphism with coronary artery disease has been explored, but the results remain controversial. Thus, a meta-analysis was conducted to provide a comprehensive estimate of this association. We selected ten articles encompassing 12 independent case-control studies with 7063 cases and 10,453 controls for this meta-analysis. Overall, we found significant associations between the *CYP2J2* G-50T polymorphism and coronary artery disease risk in three genetic models (allele model: odds ratio (OR) = 1.19, 95% confidence interval (CI) = 1.05–1.34; homozygote model: OR = 2.25, 95% CI = 1.27–4.01; recessive model: OR = 2.17, 95% CI = 1.22–3.86). In these three genetic models, a significant association was observed in Caucasians but not in Asians when the data were stratified by ethnicity. However, no significant associations were found between the *CYP2J2* polymorphism G-50T and coronary artery disease risk in heterozygote model and dominant model. In conclusion, our meta-analysis suggested that the *CYP2J2* G-50T polymorphism was associated with coronary artery disease risk in the allele, homozygote and recessive models in Caucasians.

## INTRODUCTION

Coronary artery disease (CAD) is a major cause of cardiovascular morbidity and mortality worldwide [1]. However, we do not totally understand its fundamental mechanism. As a multifaceted and polygenic illness, CAD may result from interactions between various environmental influences and genetic factors [2, 3]. Epidemiological studies have demonstrated that CAD is the consequence of several risk factors, such as age, body mass index (BMI), smoking, less exercise, gender, diabetes, hypercholesterolemia and low intake of dietary fiber. In addition to these risks, it is undeniable that hereditary factors play a crucial role. Until now, both genome-wide association studies (GWASs) and candidate gene studies have stated that numerous genetic variants are associated with susceptibility to CAD in various populations [4, 5].

Cytochrome P450 (CYP) enzyme 2J2 (CYP2J2) is one of the predominant CYP epoxygenase isoforms and is abundantly expressed in heart tissue [6]. In endothelial cells and cardiomyocytes, epoxyeicosatrienoic acids (EETs) are predominantly synthesized by CYP2J2 [7], which has been considered a vascular protective factor [8, 9]. In *CYP2J2*, the G-50T (CYP2J2-76G > T; *7 allele) polymorphism in the proximal promoter disturbs a Sp1transcription factor binding site and brings about less *CYP2J2* transcription [10]. Some previous researches have reported that the *CYP2J2* – 50T variant allele was associated with CAD risk [10, 11], but there are a few opposing published results [12–19]. In addition, a meta-analysis has been conducted to assess the relationship between the *CYP2J2*-50T polymorphism and CAD risk and had a negative result, which was not analyzed in detail [13]. Against this background, we add some new studies in our meta-analysis and present a more comprehensive analysis to evaluate the association between the *CYP2J2*-50T polymorphism and CAD risk.

## RESULTS

### Study characteristics

**Figure 1:**
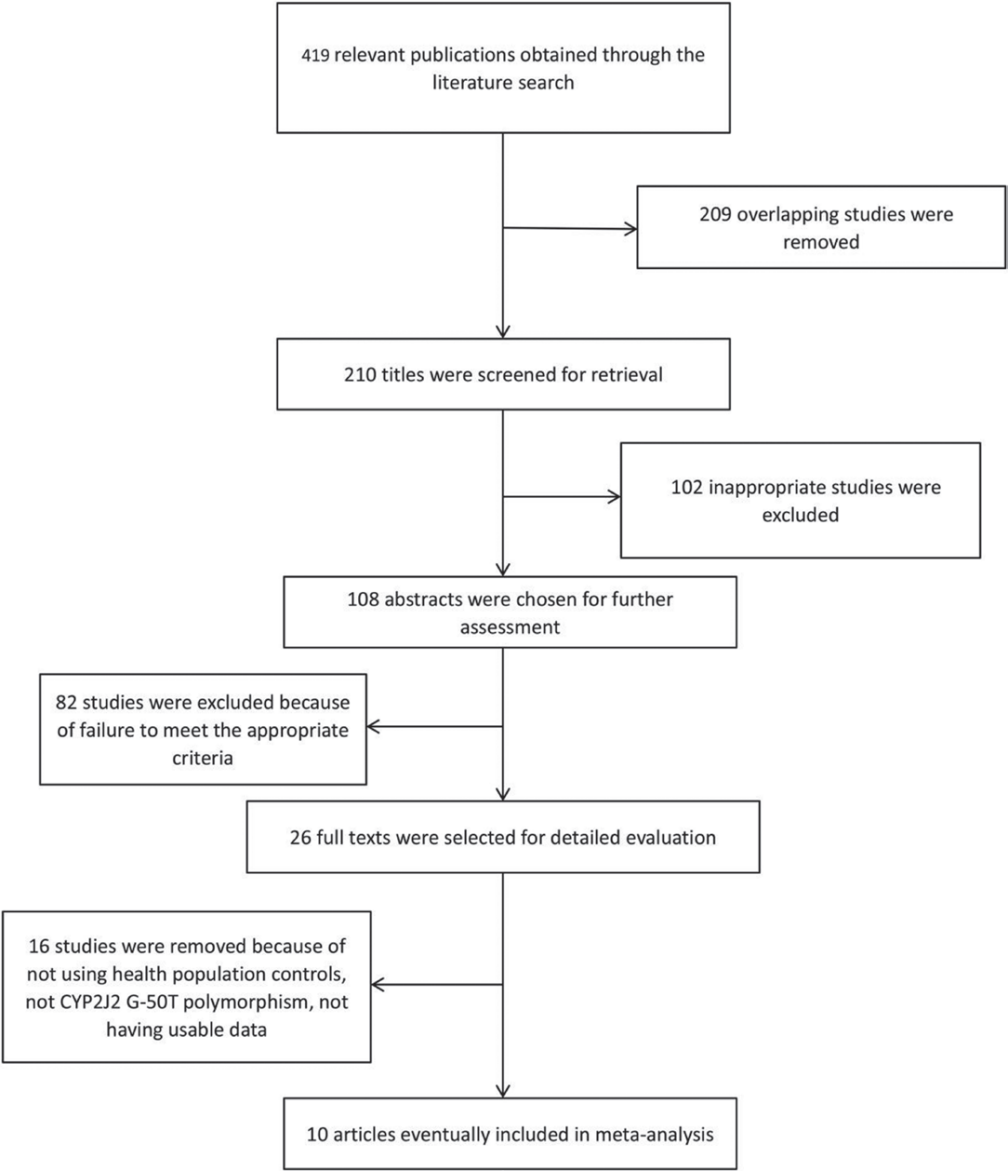
Flow diagram of studies through the meta-analysis

The process of study selection is shown in Figure 1. In total, there were 419 potentially relevant publications found through the literature search. Then, we screened the titles, abstracts and full-texts and excluded 409 articles due to unrelated research, review and data duplication. Lastly, a total of ten studies, including 7063 cases and 10,453 controls, were included in this meta-analysis. Among these publications, only two studies contained different population research. In the controls of all included studies, genotype distributions were consistent with Hardy-Weinberg equilibrium (HWE). The detailed characteristics of each study are listed in Table 1, and Table 2 shows the genotype distributions of the *CYP2J2* G-50T polymorphism in cases and controls.

**Table 1:** Characteristics of the included studies

Included study	Year	Ethnicity	Genotyping method	Source of controls	No. of case	No. of control	Quality score
Arun Kumar et al.	2015	Asians	Real-time PCR	HB	287	321	9
Tzveova et al.	2015	Caucasians	Taqman	PB	254	470	10
Zhu et al.	2013	Asians	Taqman	PB	573	455	11
Zhu et al.	2013	Asians	Taqman	PB	286	138	11
Xu et al.	2011	Asians	Taqman	HB	1344	1267	9
Fava et al.	2010	Caucasians	Taqman	PB	132	5608	9
Lee et al.	2007	Caucasians	MALDI TOF MS	PB	731	566	10
lee et al.	2007	African-American	MALDI TOF MS	PB	211	268	10
Hoffmann et al.	2007	Caucasians	PCR	PB	2547	696	11
Liu et al.	2007	Asians	PCR	HB	200	200	9
Lung et al.	2006	Asians	PCR	HB	209	209	9
Spiecker et al.	2004	Caucasians	PCR	HB	289	255	10

PB: Population-based; HB: Hospital-based; MALDI TOF MS: matrix-assisted laserdesorption/ionization time-of-flight mass spectrometry; PCR, polymerase chain reaction.

**Table 2:** The genotypes distribution and allele frequencies of eligible studies

Included study	Ethnicity	group	genotype			Allele frequencies (%)		HWE(*P*)
			GG	GT	TT	G	T	
Arun Kumar et al.	Indian	case	251	34	2	93.4	6.6	0.99
		control	286	34	1	94.4	5.6	
Tzveova et al.	Bulgarian	case	217	32	5	91.7	8.3	0.68
		control	428	50	2	94.3	5.7	
Zhu et al.	Han	case	521	51	1	95.4	4.6	0.28
		control	411	44	0	95.2	4.8	
Zhu et al.	Uygur	case	253	32	1	94.1	5.9	0.56
		control	125	13	0	95.3	4.7	
Xu et al.	China	case	1220	118	6	95.2	4.8	0.56
		control	1147	116	4	95.1	4.9	
Fava et al.	Swedes	case	112	19	1	92.0	8.0	0.33
		control	4760	819	29	92.2	7.8	
Lee et al.	Caucasian	case	648	83		93.6	6.4	> 0.05
		control	501	65				
lee et al.	African-American	case	167	44		84.5	15.5	> 0.05
		control	189	79				
Hoffmann et al.	Germany	case	2225	313	9	93.5	6.5	0.83
		control	618	76	2	94.3	5.7	
Liu et al.	China	case	136	56	8	82.0	18.0	0.45
		control	156	40	4	88.0	12.0	
Lung et al.	China	case	187	22	0	94.7	5.3	0.52
		control	191	18	0	95.7	4.3	
Spiecker et al.	Germany	case	239	43	7	90.1	9.9	0.78
		control	228	26	1	94.5	5.5	

HWE(*P*), the *P*-values of the Hardy-Weinberg equilibrium test of control group.

### Results of the meta-analysis

**Figure 2:**
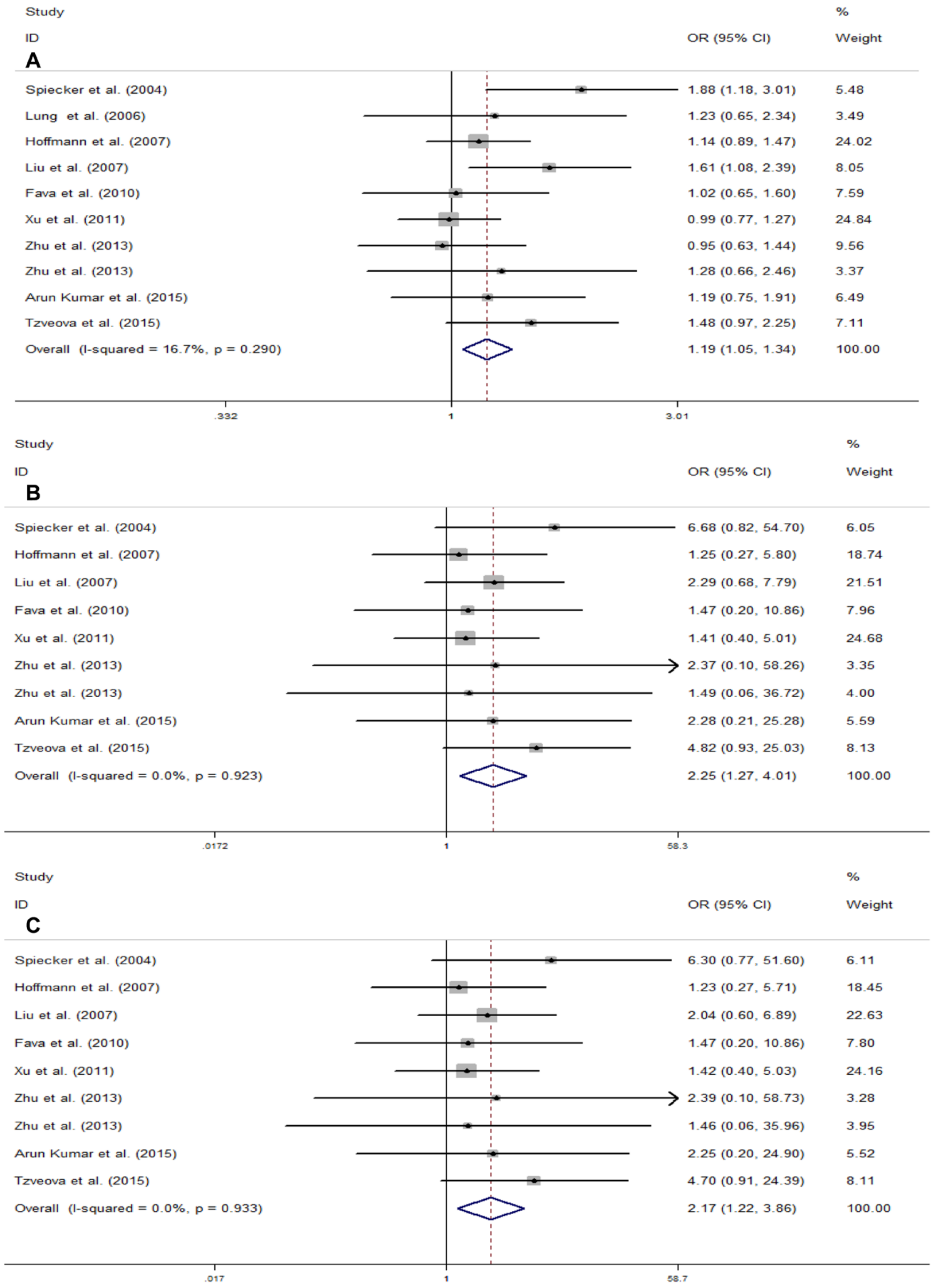
Meta-analysis for the association of CYP2J2 G-50T polymorphism and CAD risk in total population (**A**) Allele genetic model (T vs.G); (**B**) Homozygote genetic model (TT vs. GG); (**C**) Recessive genetic model (TT vs. GT + GG). For each study, the estimation of OR and its 95% CI are plotted with a box and a horizontal line. ◊, pooled ORs and its 95% CIs.

The main results of the meta-analysis are summarized in Table 3. The study by Lee *et al*. was excluded in the pooled analysis of all genetic models, except for the dominant model, because of the unknown rate of the mutational homozygote (TT) in case and control groups. Additionally, as a result of the absence of the mutational homozygote (TT) in both case and control groups, the study by Lung *et al*. was excluded in the homozygote genetic model and the recessive model. Overall, no significant association was found between the *CYP2J2* G-50T polymorphism and CAD risk in two genetic models (heterozygote model, OR = 1.13, 95% CI = 0.99–1.28, *P* = 0.071; dominant model, OR = 1.09, 95% CI = 0.97–1.22, *P* = 0.137). However, a different result was obtained in the next analysis. The risk was significantly altered for the *CYP2J2* G-50T polymorphism and CAD in the remaining three comparisons (T vs. G, OR = 1.19, 95% CI = 1.05–1.34, *P* < 0.01; TT vs. GG, OR = 2.25, 95% CI = 1.27–4.01, *P* < 0.01; TT vs. GT + GG, OR = 2.17, 95% CI = 1.22–3.86, *P* < 0.01) (Figure 2).

**Table 3:** The main results of this meta-analysis

Genotype contrast	population	Sample size		Type of model	Number of studies	Test of association			Heterogeneity	
		case	control			OR	95% CI	*P* value	*I*^2^	*P* value
T vs. G	Over all	6121	9619	Fixed	10	1.19*	1.05–1.34*	< 0.01	16.7%	0.290
TT vs. GG	Over all	5912	9410	Fixed	9	2.25*	1.27–4.01*	< 0.01	0.0%	0.923
GT vs. GG	Over all	6121	9619	Fixed	10	1.13	0.99–1.28	0.071	0.0%	0.661
TT vs. GT + GG	Over all	5912	9410	Fixed	9	2.17*	1.22–3.86*	< 0.01	0.0%	0.933
TT + GT vs. GG	Over all	7063	10453	Fixed	12	1.09	0.97–1.22	0.137	34.1%	0.117

OR: odds ratio; *OR with statistical significance.

### Subgroup analysis results

**Figure 3:**
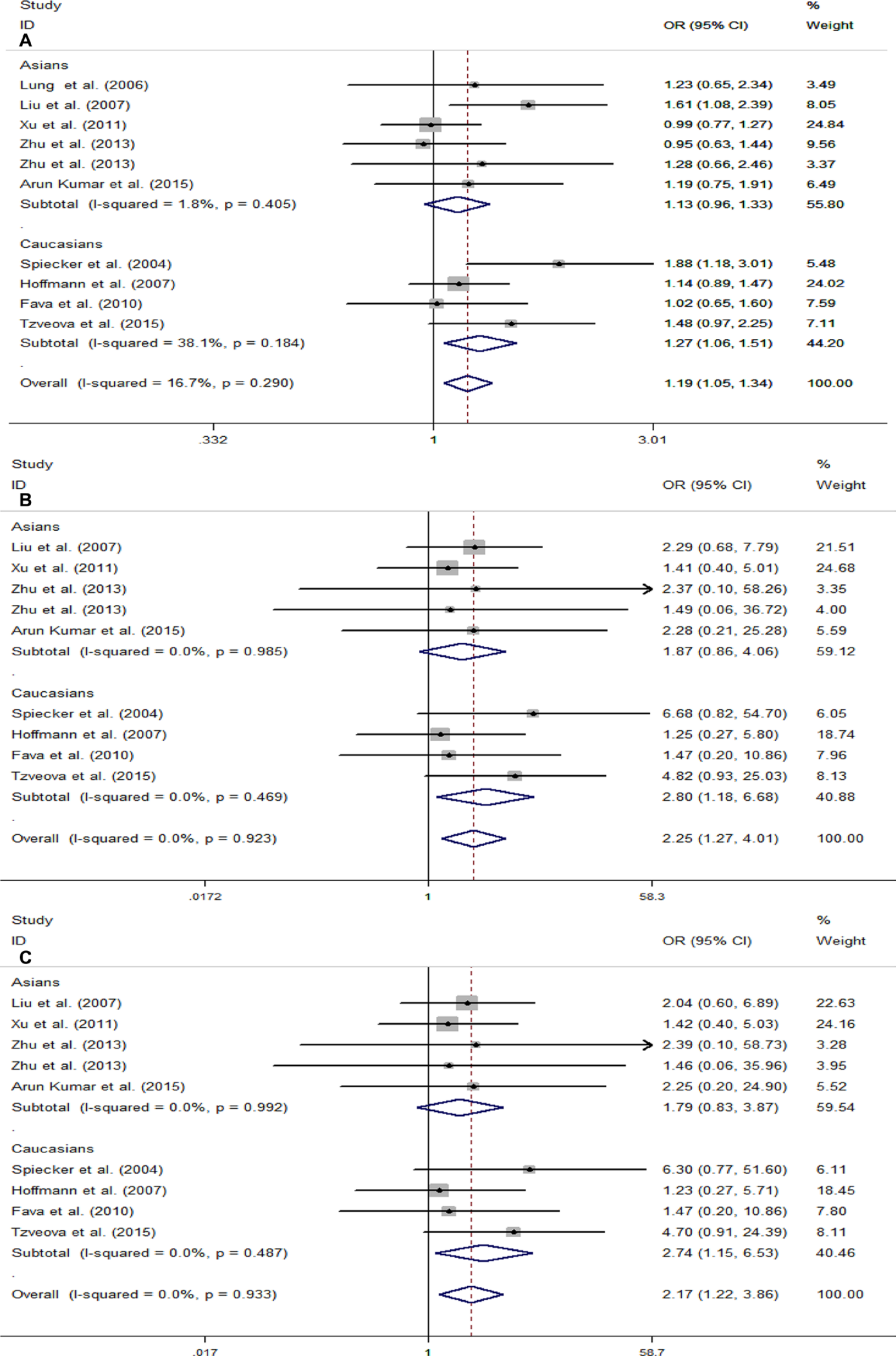
Forest plots for stratification study of the association between CYP2J2 G-50T polymorphism and CAD risk under the three genetic models (**A**) T vs. G; (**B**) TT vs. GG; (**C**) TT vs. GG/GT. For each study, the estimation of OR and its 95% CI are plotted with a box and a horizontal line. ◊, pooled ORs and its 95% CIs.

To investigate whether the association between *CYP2J2* G-50T polymorphism and CAD varies among different populations, we performed subgroup analysis based on the region of origin of the study population. In the subgroup analyses of ethnicity, a significant association was observed in Caucasians under the above three genetic models (T vs. G, OR = 1.27, 95% CI = 1.06–1.51, *I*^2^ = 38.1%; TT vs. GG, OR = 2.80, 95% CI = 1.18–6.68, *I*^2^ = 0.0%; TT vs. GT + GG, OR = 2.74, 95% CI = 1.15–6.53, *I*^2^ = 0.0%) (Figure 3). In contrast, we did not find any significant relationship between *CYP2J2* G-50T polymorphism and CAD in Asians (Figure 3).

### Test of heterogeneity

According to *P* values of the Chi-Square-based Q-statistical test, no significant heterogeneity was detected between studies in all genetic models (Table 3). Therefore, we used a fixed-effects model for pooled analysis in all genetic models.

### Sensitivity analysis and publication bias

**Figure 4:**
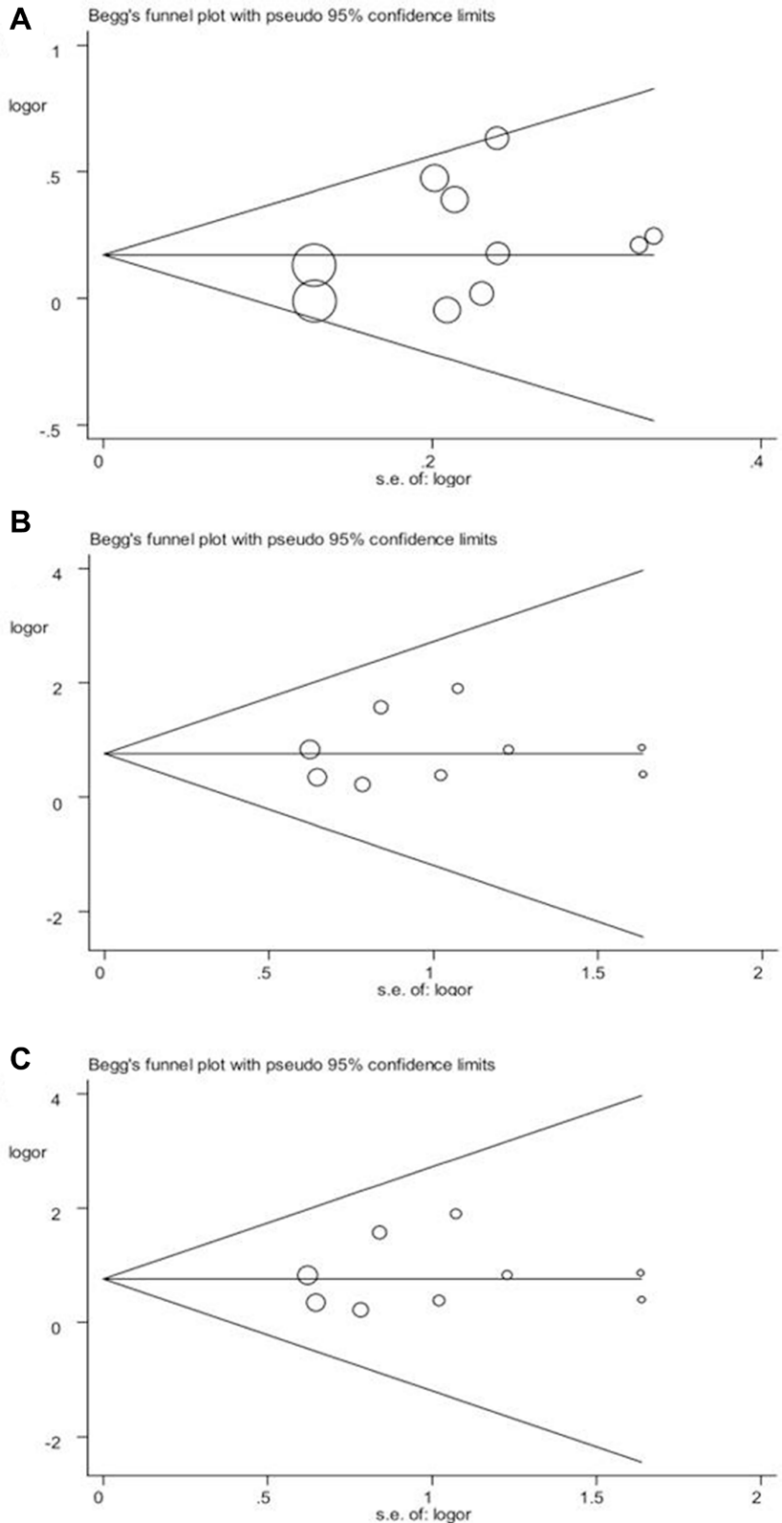
Begg's funnel plot of publication bias in the meta-analysis of the association of CYP2J2 G-50T polymorphism and CAD risk under three genetic models (**A**) T vs. G; (**B**) TT vs. GG; (**C**) TT vs. GG/GT. Each point represents a separate study for the indicated association.

The stability of the overall results was also assessed by sequential omission of individual studies. Sensitivity analysis demonstrated the reliability and stability of our results because the combined results were not significantly influenced by any individual study. We used funnel plots to detect publication bias and the shape of the Begg 's funnel plot found no obvious asymmetry, which suggests the absence of publication bias in the overall meta-analysis (Figure 4). The results of Egger's test were as follows: (allele: *P* = 0.218; homozygous: *P* = 0.632; recessive: *P* = 0.635), which further provided no evidence of publication bias.

## DISCUSSION

Like other CYP2 family genes, CYP2J2 contains nine exons and eight introns and spans ~40.3 kb on human chromosome 1, band p31.3–p31.2 [20]. EETs, synthesized by CYP2J2, have many important physiological actions including maintaining cardiomyocyte viability suffering from damaging stimuli and enhance the recovery of cells from oxygen deprivation [21]. The G-50T (also called rs890293) polymorphism is one of the most relevant polymorphisms associated with CAD according to polymorphism frequency and functional importance [22]. Decreasing quantity of CYP2J2 protein may affect EETs generation and then subsequently increase the incidence of CAD. The study by Spiecker *et al*. has proven that the G-50T polymorphism can lead to less *CYP2J2* gene transcription by disturbing a Sp1 transcription factor binding site [10]. Several studies have aimed at investigating the relationship between the *CYP2J2* G-50T polymorphism and CAD risk and have found there is an association between them [10, 11]. Conversely, other researches [12–19] also had comprehensive studies of the role of the *CYP2J2* G-50T polymorphism in the development of CAD risk and found no significant association between the *CYP2J2* G-50T polymorphism and CAD susceptibility, which indicates that the development of risk of CAD may not be affected by variant alleles.

Therefore, a meta-analysis containing 10 case-control studies with 7063 cases and 10,453 health controls was performed to comprehensively evaluate the effect. As far as we know, the result of a previous meta-analysis showed that there was no significant association between *CYP2J2* G-50T polymorphism and CAD risk in dominant model comparisons (OR = 1.12, 95% CI = 0.95–1.31) and additive model comparisons (OR = 1.15, 95% CI = 0.97–1.36) [13]. However, the study carried out by Xu *et al*. did not offer any detailed data in the meta-analysis and did not comprehensively evaluate the association in all comparisons. Thus, we subsequently added some new eligible researches into our more complete meta-analysis to extensively investigate the role of the *CYP2J2* G-50T polymorphism in the development of CAD. Our study suggested that *CYP2J2* G-50T polymorphism was not associated with CAD risk under the dominant model and heterozygote model. However, there was a significant relationship between the *CYP2J2* G-50T polymorphism and CAD risk in other genetic models (T vs. G, OR = 1.19, 95% CI = 1.05–1.34; TT vs. GG, OR = 2.25, 95% CI = 1.27–4.01; TT vs. GT + GG, OR = 2.17, 95% CI = 1.22–3.86). In addition, the stratification analyses of studies by ethnicity found that the *CYP2J2* G-50T polymorphism in Caucasians was significantly associated with an increased risk for CAD in the three genetic models. Nevertheless, we did not found any association of the polymorphism with CAD risk in the Asian population, and these results suggest that the SNP may be an ethnicity-dependent factor in CAD progression. Besides, the significant association only can be found under the allele model, recessive model and homozygote model, which suggested that the rare TT genotype may play a crucial role. To clarify inconsistence between our consequence and previous meta-analysis result, we performed a comparison and found a few reasons. First, previous study did not comprehensively evaluate the association in all comparisons and might ignore some association in a part of all genetic models. Second, we added new eligible researches and omitted unqualified articles when we perform a meta-analysis in some genetic models.

Some limitations of this meta-analysis should be addressed. Because no adjustment for other covariates was performed, our analyses based on unadjusted OR values may accordingly have low power when we estimated the real association. As the number of the included studies might be insufficient, some stratification analyses do not have enough statistical power to identify the effect. Environment factors may influence the association of the *CYP2J2* G-50T polymorphism with CAD risk through a gene-environment interaction. However, we did not evaluate the potential gene-environment effect in this study because of the unavailability of the original data.

In conclusion, our meta-analysis found that the *CYP2J2* G-50T polymorphism among Caucasians was associated with CAD risk in certain genetic models, such as the allele model, recessive model and homozygote model. Thus, we can speculate that in Caucasians, the rare TT genotype may play an important role in the progression of CAD. However, more studies based on larger sample sizes are required to validate the present findings in the future.

## MATERIALS AND METHODS

### Study identification

Two authors independently conducted a systematic literature search of the PubMed, Elsevier, China National Knowledge Infrastructure platform and Wanfang databases to identify studies of the relationship between *CYP2J2* G-50T polymorphism and CAD. To identify all possible studies as comprehensively as possible, we used the following various keywords: “*CY*P2J2”, “polymorphism,” “variant,” “G-50T polymorphism,” “-76G > T polymorphism”, “rs890293,” “coronary artery disease,” “CAD,” “coronary heart disease,” and “CHD.” Additional studies were selected by searching references cited manually in the appropriate articles. The literature search was finally performed on April 25, 2017.

### Inclusion criteria

Studies included in this meta-analysis had to meet the following criteria: (a) case control studies of the association of the *CYP2J2* G-50T polymorphism with CAD risk; (b) subjects in the case group had confirmed diagnoses; (c) genotype frequencies for both cases and controls were available; and (d) the distribution of genotypes in the control group was consistent with HWE. If there were numerous articles from the same study, we selected the most relevant one in our analysis. Case reports and reviews were excluded.

### Data extraction

Information was independently extracted by two authors and disagreement was addressed by discussion between them. From each included study, we extracted the following information: the author's first name, publication year, country, ethnicity of the study population, source of controls, genotyping methods, sample size of cases and controls, genotype distribution of the *CYP2J2* G-50T polymorphism in cases and controls, and HWE of the control group. Quality of studies was assessed according to the predefined criteria based on previous observational studies (Table 4) [23, 24].

**Table 4:** Scale for quality assessment

Criteria	Score
**Representativeness of cases**	
selected from case population with clearly defined sampling frame	2
selected from case population without clearly defined sampling frame or with extensive inclusion/exclusion criteria	1
No method of selection described	0
**Credibility of controls**	
Population-based	3
Blood donors or volunteers	2
Hospital-based	1
Not described	0
**Ascertainment of CAD**	
Clearly described objective criteria for diagnosis of CAD, histological confirmation	2
Diagnosis of CAD by patient self-report or by patient history	1
Not described	0
**Genotyping examination**	
Genotyping done under blinded condition	1
Not mentioned	0
**Hardy-Weinberg equilibrium**	
Equilibrium in controls	2
Disequilibrium in controls	1
No checked	0
**Association assessment**	
Assess association between genotypes and CAD with appropriated statistics and adjustment for confounders	2
Assess association between genotypes and CAD with appropriated statistics without adjustment for confounders	1
Inappropriate statistics used	0

### Statistical analysis

We assessed the strength of association of *CYP2J2* G-50T polymorphism with CAD risk with odds ratios (ORs) and 95% confidence intervals (CIs) in the allele model (T vs. G), heterozygote model (GT vs. GG), homozygote model (TT vs. GG), recessive model (TT vs. GG + GT) and dominant model (TT + GT vs. GG). The significance of combined ORs was determined by using the *Z*-test. The *Q* test was used to evaluate the heterogeneity between the included studies. If *P* > 0.05, which indicated no significant heterogeneity, we chose the fixed-effects model (Mantel-Haenszel) to combine the data; if not, the random-effects model (DerSimonian-Laird) was applied. Subgroup analyses were performed according to ethnicity. We detected publication bias by using Begg's funnel plots and Egger's test. The stability of results was assessed by a sensitivity analysis performed by sequential omission of individual studies. The χ^2^-test was used to check the HWE of genotype distribution in the control group. All the tests were two-sided and *P* < 0.05 was considered statistically significant. The data analyses were performed using STATA v12.0 software (Stata Corporation, College Station, TX, USA).
